# Ansa Pancreatica: A Rare Culprit in Recurrent Acute Pancreatitis

**DOI:** 10.7759/cureus.59235

**Published:** 2024-04-28

**Authors:** Manjeet K Goyal, Ashita R Vuthaluru, Varna Taranikanti

**Affiliations:** 1 Gastroenterology and Hepatology, Dayanand Medical College and Hospital, Ludhiana, IND; 2 Anesthesia and Critical Care, All India Institute of Medical Sciences, New Delhi, IND; 3 Foundational Medical Studies, Oakland University William Beaumont School of Medicine, Rochester, USA

**Keywords:** clinical diagnosis, pancreatic ductal anomalies, recurrent acute pancreatitis, ansa pancreatica, extrahepatic biliary tract

## Abstract

Ansa pancreatica is a rare anatomical variation of the pancreatic duct system, characterized by a reversed S-shaped loop that connects the main pancreatic duct to the minor papilla. Its clinical significance, particularly with recurrent acute pancreatitis, has been a subject of interest, but remains poorly understood due to the rarity of reported cases. We report the case of a 34-year-old female with a history of multiple episodes of acute pancreatitis, diagnosed with ansa pancreatica using magnetic resonance cholangiopancreatography (MRCP). The patient presented with severe epigastric pain radiating to the back, accompanied by vomiting and nausea. Laboratory findings revealed elevated serum amylase and lipase levels. MRCP imaging identified the ansa pancreatica, showing a distinct looping ductal variant terminating near the minor papilla. The patient underwent successful endoscopic treatment with significant improvement in symptoms and no recurrence of pancreatitis at follow-up.

In this case, the presence of ansa pancreatica underscores the variant's potential role in predisposing individuals to recurrent episodes of acute pancreatitis. The literature review highlights the anatomical description of ansa pancreatica and its speculated pathophysiological mechanism leading to pancreatitis, primarily due to impaired pancreatic juice drainage. Despite its rarity, recognizing ansa pancreatica is crucial for the appropriate management of idiopathic recurrent pancreatitis, especially in cases where conventional causes are absent. This case report and literature review emphasize the importance of considering ansa pancreatica in the differential diagnosis of recurrent acute pancreatitis. Further research is needed to elucidate the exact mechanism by which this anatomical variant contributes to pancreatitis and to explore potential therapeutic interventions. Awareness and early detection of ansa pancreatica can lead to targeted treatments, reducing the morbidity associated with recurrent pancreatitis episodes.

## Introduction

Recurrent acute pancreatitis (RAP) is defined as a pathological condition of having more than two episodes of acute pancreatitis without evidence of chronic pancreatitis with complete resolution of inflammation for more than three months between attacks [1]. The etiology of RAP is diverse, encompassing gallstones, alcohol abuse, metabolic disorders, and anatomical variations of the pancreatic ductal system interfering with pancreatic juice outflow, among others. Certain ductal anomalies, such as peri-ampullary diverticula, duct ectasias, and pancreatic divisum, are also responsible for RAP [2]. Despite advancements in diagnostic modalities, a significant proportion of RAP cases remain idiopathic, posing a challenge to clinicians in terms of management and prevention of recurrence [1]. Ansa pancreatica is a rare anatomical variant of the pancreatic duct system, first described by Dawson and Langman. It is characterized by an abnormal looping of the pancreatic duct, where a branch descends as an extension of the main pancreatic duct, later ascending to form a loop that terminates at the minor papilla [3,4]. This variant has been implicated in the pathogenesis of RAP in a handful of case reports; hence, it is difficult to establish a concrete causal association [5]. In this article, we report a case of RAP in a 34-year-old Indian woman, with ansa pancreatica being the most likely etiology. Further, we developed a pattern of the different embryological ductal variants that can cause RAP and may be of clinical aid to physicians and surgeons while planning the management of these cases. 

## Case presentation

A 34-year-old woman (IRB-20/189; following Helsinki declaration) with multiple episodes of acute pancreatitis of unidentified cause arrived at the gastroenterology emergency department with vomiting and excruciating epigastric pain for more than six hours. There was a history of multiple episodes of similar symptoms in the past. There was no significant family history or past medical drug or substance use. Blood analysis revealed high serum levels of lipase and amylase (2614 and 3723, respectively, with normal values <100 IU/ml) with normal levels of serum triglycerides, IgG4, and anti-nuclear antibodies (see Table 1).

**Table 1:** Results of the laboratory investigations

Laboratory parameter	Value	Reference range
Hemoglobin	11.5 g/dL	11.5-12.5 g/dL
Leukocyte count	6500/mm^3^	4000-11,000/mm^3^
Platelet count	333,000/mm^3^	150,000-550,000/mm^3^
Bilirubin	0.68 mg/dL	<1 mg/dL
Alanine transaminase	29 IU/L	<40 IU/L
Aspartate transaminase	33 IU/L	<40 IU/L
Alkaline phosphatase	112 IU/L	40-129 IU/L
Blood urea nitrogen	13 mg/dL	10-20 mg/dL
Serum creatinine	0.73 mg/dL	<1 mg/dL
Amylase	2614 IU/mL	<100 IU/mL
Lipase	3723 IU/mL	<100 IU/mL
Serum triglyceride	112 mg/dL	<150 mg/dL
IgG4 level	1.5 g/L	<2 g/L

**Figure 1:**
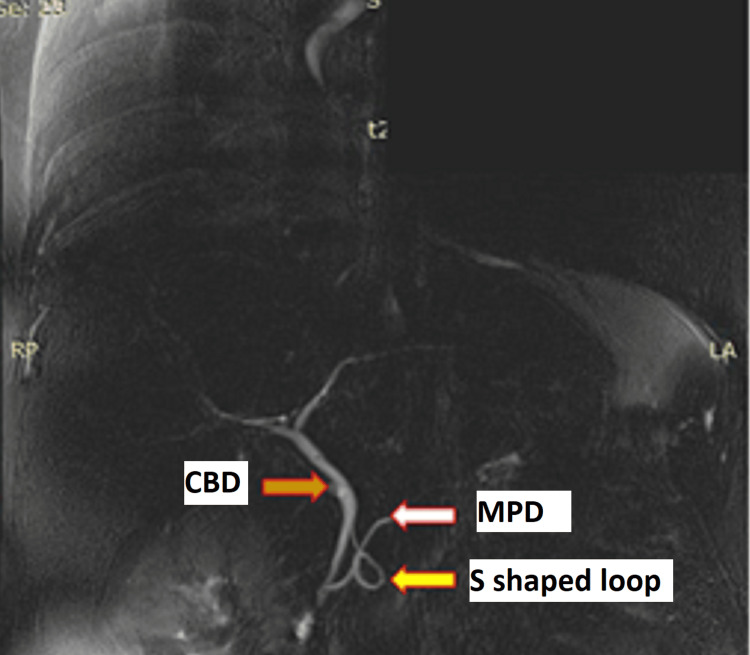
MRCP depicting pancreatic and biliary ductal system The blue arrow indicates CBD; the white arrow shows MPD; the yellow arrow depicts an S-shaped loop connecting the major and minor pancreatic duct. CBD - common bile duct; MPD - major pancreatic duct; MRCP - magnetic resonance cholangiopancreatography

Radiological investigations were negative for gallstones or considerable biliary dilation on ultrasonography. Ultrasonography was suggestive of a bulky pancreas. A preliminary diagnosis of acute pancreatitis was considered, and she was managed with IV fluids, analgesics, and other supportive measures. Her symptoms gradually subsided with the normalization of biochemical parameters over the next few days. After the inflammation had subsided (asymptomatic with normalization of amylase, lipase, and inflammatory markers), a magnetic resonance cholangiopancreatography (MRCP) was performed, which revealed an S-shaped loop connecting the duct of Santorini (accessory pancreatic duct) with the duct of Wirsung (main pancreatic duct), indicating ansa pancreatica with dilatation of the main pancreatic duct to 3 mm in the tail region (Figure 1). An endoscopic retrograde cholangiopancreatography (ERCP) confirmed MRCP findings, and sphincterotomy was done as a therapeutic measure. The patient is currently under follow-up for more than three years and is in good health with no further complications.

## Discussion

**Figure 2:**
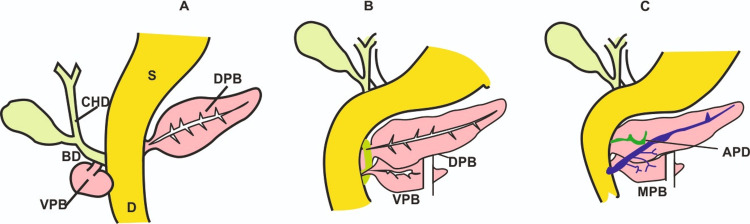
The illustrations depict the MPD formed by the anastomosis of the duct of distal two-thirds of the DPB and the duct of the entire VPB A: During the eighth week of embryogenesis, the ventral and the dorsal pancreatic ducts arise from the Foregut. B: Migration of ventral pancreatic bud towards dorsal pancreatic bud; C: Fusion of dorsal pancreatic bud with ventral pancreatic bud resulting in the formation of the main pancreatic duct (duct of Wirsung), which connects to the second part of the duodenum. The accessory duct is formed from the ventral pancreatic duct and provides an alternate route for pancreatic secretions to enter the duodenum. MPD - main pancreatic duct; S - stomach; GB - gall bladder; D - duodenum; CHD - common hepatic duct; BD - bile duct; VPD - ventral pancreatic bud; DPB - dorsal pancreatic bud; APD - accessory pancreatic duct Image credit: Varna Taranikanti

**Figure 3:**
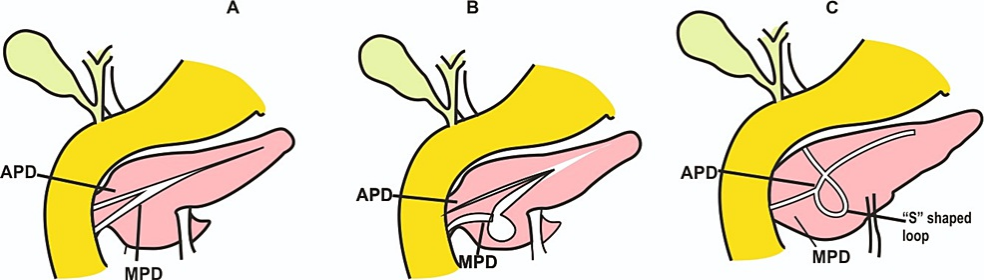
The illustrations depict the ansa pancreatica formed from the proximal part of APD that loops in a S-shaped manner A: Normal embryogenesis of VPD and MPD. B: Looping/kinking of MPD. C: S-shaped loop (ansa pancreatica) connecting MPD with APD; failure of normal fusion between the ventral and dorsal pancreatic ducts leads to the formation of an S-shaped loop where there is kinking of the main pancreatic duct and connection of MPD with APD through the loop. APD - accessory pancreatic duct; MPD - main pancreatic duct; VDP - ventral pancreatic bud Image credit: Varna Taranikanti

The ventral and the dorsal pancreatic buds develop from the endodermal diverticula of the duodenum and grow into the ventral and dorsal mesentery, respectively. During the fifth week of fetal development, the ventral bud migrates around the posterior side of the duodenum to fuse with the dorsal pancreatic bud [6]. The main pancreatic duct (duct of Wirsung), which opens at the major duodenal papilla, is formed by the anastomosis of the distal two-thirds of the dorsal pancreatic duct and the entire ventral pancreatic duct (Figure 2) [7]. The proximal part of the dorsal pancreatic duct sometimes disappears, but when it persists, it forms the accessory pancreatic duct (duct of Santorini) that opens at the minor duodenal papilla [8]. Ansa pancreatica is a scarce variety of ductal anomalies in which the major pancreatic duct (duct of Wirsung) joins the minor pancreatic duct (duct of Santorini) in an S-shaped loop (Figure 3).

Ansa pancreatica is characterized by an abnormal looping of the pancreatic duct, where a branch descends as an extension of the main pancreatic duct, later ascending to form a loop that terminates at the minor papilla [4]. This anatomical anomaly can hinder the drainage of pancreatic secretions, potentially leading to pancreatitis. Despite its significance, ansa pancreatica remains underreported, primarily due to its rarity and the subtlety of its presentation [9].

The case of the 34-year-old Indian female underscores the diagnostic challenge posed by ansa pancreatica. The patient presented with recurrent episodes of acute pancreatitis, with no apparent etiology identified through conventional diagnostic modalities such as abdominal ultrasonography and CT scans. Only conducting MRCP revealed the presence of ansa pancreatica, demonstrating the critical role of advanced imaging techniques in diagnosing this condition. Management of RAP due to ansa pancreatica involves addressing the underlying anatomical anomaly to restore normal pancreatic duct drainage. In this case, endoscopic intervention, specifically minor papilla sphincterotomy, was employed to alleviate the obstruction caused by the ansa pancreatica loop. Furthermore, the timely identification and endoscopic treatment led to the alleviation of symptoms and prevention of chronic pancreatitis, a well-known complication of RAP. This approach aligns with the recommendations emphasizing the potential benefits of endoscopic therapies in mitigating the recurrence of acute pancreatitis associated with pancreatic ductal anomalies [1].

However, the decision to proceed with endoscopic intervention must be carefully considered, given the risk of complications such as post-ERCP pancreatitis. The risk-benefit ratio should be thoroughly evaluated, taking into account the patient's clinical presentation, the severity of symptoms, and the presence of other risk factors for pancreatitis [10].

An extensive search of Pubmed yielded only a handful of cases (Tables 2 and 3). Most of these cases have been diagnosed incidentally and were diagnosed either post-operatively, cadaverically, or radiologically in patients presenting with acute pancreatitis. The present case contributes to the growing body of literature on ansa pancreatica as a cause of RAP, highlighting the importance of a high index of suspicion for this rare anatomical variant in patients with idiopathic RAP. It underscores the need for comprehensive diagnostic evaluation, including advanced imaging techniques like MRCP, to accurately identify ansa pancreatica. Furthermore, to the best of our knowledge, this is the first case of RAP with ansa pancreatica in a female, of childbearing age where gallstones and microlithiasis are the predominant causes. Our case is also unique because it is the first case reported of a female from the Indian subcontinent, while other cases reported were from the West. Moreover, this is the only case report in the literature that describes a follow-up of more than three years with no further attacks of acute pancreatitis. Therefore, it emphasizes the potential role of endoscopic interventions in managing RAP attributed to this condition, while also calling for caution due to the associated risks.

**Table 2:** Studies reporting incidence of ansa pancreatica ERCP - endoscopic retrograde cholangiopancreatography; MRCP - magnetic resonance cholangiopancreatography

Authors (year)	Subjects examined	Methods of subject examination	Country	Incidence of ansa pancreatica
Langman, Dawson (1961) [4]	120	Cadaveric and radiologic	Canada	17%
Kamisawa et al. (1998) [6]	213	ERCP	Japan	13.6%
Adibelli et al.(2016) [11]	1158	MRCP	Turkey	1.2%
Hayashi et al. (2016) [5]	660	MRCP	Japan	1.2%
Narayanan, Shabna (2017) [12]	50	Cadaveric	India	2%
Liessi et al. (2010) [13]	300	Secretin enhanced MRCP	Italy	1%

**Table 3:** Summary of various case reports on ansa pancreatica ERCP - endoscopic retrograde cholangiopancreatography; MRCP - magnetic resonance cholangiopancreatography

Authors	Age	Sex	Presentation	Diagnostic modality	Treatment modality	Outcome
Jarrar, et al. [3]	53	Male	Acute pancreatitis	Post-operative cholangiography	None	-
Hussain, et al. [14]	11	Male	Acute pancreatitis	MRCP	Sphincterotomy	Resolution
Shaikh, et al. [15]	49	Female	Acute pancreatitis	Endoscopic ultra-sonography	Sphincterotomy	Resolution
Guerrom, et al. [16]	24	Male	Acute pancreatitis	MRCP	Sphincterotomy	Resolution
Ismail, et al. [17]	32	Female	Acute pancreatitis	MRCP	None	-
Bhasin, et al. [18]	21	Male	Severe acute pancreatitis	ERCP	Surgery	Resolution
Chahine, et al. [19]	67	Female	Acute pancreatitis	Operative	Surgery for cholecystectomy	Resolution
Bakouri, et al. [20]	44	Female	Acute pancreatitis	MRCP	Sphincterotomy	Resolution
Present case	34	Female	Recurrent acute pancreatitis	MRCP	Sphincterotomy	Resolution

In conclusion, ansa pancreatica represents a rare but significant cause of RAP, necessitating awareness among clinicians to ensure timely diagnosis and appropriate management. Future research should focus on elucidating the pathophysiological mechanisms underlying RAP in patients with ansa pancreatica and optimizing treatment strategies to prevent recurrence and improve patient outcomes.

## Conclusions

Ansa pancreatica is a rare cause of RAP. Therefore, an awareness of these anomalies helps in surgical planning, which could improve the quality of life and reduce the disability-adjusted life years of such patients. 
